# Deciphering HPV‐Associated Immune Evasion in Cervical Cancer Through Multi‐Omics Profiling and Computational Screening

**DOI:** 10.1155/ancp/6756766

**Published:** 2026-08-24

**Authors:** Rui Guo, Wenting He, Xia Liu, Na Zhang

**Affiliations:** ^1^ Department of Dermatology, People’s Hospital of Ningxia Hui Autonomous Region, Ningxia Medical University, Yinchuan, China, nxmu.edu.cn; ^2^ Health Management Center, People’s Hospital of Ningxia Hui Autonomous Region, Ningxia Medical University, Yinchuan, China, nxmu.edu.cn; ^3^ Department of Obstetrics and Gynecology, People’s Hospital of Ningxia Hui Autonomous Region, Ningxia Medical University, Yinchuan, China, nxmu.edu.cn

**Keywords:** cervical neoplasms, human papillomavirus infections, IFNGR1, immune evasion, single-cell analysis

## Abstract

**Background:**

Human papillomavirus (HPV) infection is a major contributor to cervical cancer (CC), yet the molecular mechanisms driving HPV‐associated immune evasion remain largely undefined.

**Methods:**

Bulk RNA‐seq (The Cancer Genome Atlas [TCGA]–CESC) and single‐cell RNA‐seq datasets (GSE171894, GSE197461) were analyzed to elucidate transcriptional and immune landscape differences between HPV‐positive and HPV‐negative cervical tumors. Differentially expressed genes (DEGs) were identified using DESeq2. Functional enrichment analyses were conducted through gene set enrichment analysis (GSEA), gene ontology (GO), and Kyoto Encyclopedia of Genes and Genomes (KEGG) methodologies. Immune evasion feature genes were selected employing LASSO, Random Forest, and SVM‐RFE techniques. Regulatory networks for transcription factors and miRNAs were constructed. Immune infiltration was evaluated using CIBERSORT and ssGSEA. Validation of key signature genes was performed in CC cell lines (HeLa, SiHa, C33A, and W12) via real‐time quantitative polymerase chain reaction (RT‐qPCR) and Western blot. The functional roles of IFNGR1 were examined through siRNA‐mediated knockdown, complemented by CCK‐8, colony formation, Transwell migration/invasion, and flow cytometry apoptosis assays.

**Results:**

A total of 6266 DEGs effectively differentiated HPV‐positive from HPV‐negative tumors. HPV‐positive tumors exhibited enrichment in viral infection and immune response pathways, while HPV‐negative tumors demonstrated activation of oncogenic signaling. Machine learning algorithms identified IFNGR1, TRADD, and PSMB9 as immune evasion feature genes associated with HPV. Regulatory network analysis emphasized IRF1/IRF2 and several miRNAs as critical modulators. Immune infiltration analysis indicated increased infiltration of Dendritic and Plasma cells in HPV‐positive tumors, correlating with TRADD expression. Kaplan‐Meier analysis further showed a trend toward worse overall survival among HPV‐positive patients with high IFNGR1 expression (hazard ratio [HR] = 1.78, 95% confidence interval [CI]: 0.86–3.68; log‐rank *p* = 0.113). Notably, IFNGR1 was significantly upregulated in HPV‐positive CC cell lines at both mRNA and protein levels. IFNGR1 knockdown markedly inhibited proliferation, colony formation, migration, and invasion, while enhancing apoptosis in HeLa and SiHa cells, thereby confirming its essential role in the progression of HPV‐associated CC.

**Conclusion:**

This study identified IFNGR1 as a key immune evasion‐related gene in HPV‐positive CC, elucidating its regulatory network and functional contributions, while positioning it as a potential therapeutic target for HPV‐associated tumors.

## 1. Introduction

Cervical cancer (CC) remains one of the most prevalent malignancies affecting women globally, characterized by high morbidity and mortality rates, particularly in low‐ and middle‐income countries [1, 2]. Persistent infection with high‐risk human papillomavirus (HR‐HPV), especially HPV16 and HPV18, is recognized as the primary etiological driver of cervical carcinogenesis, with ~99.7% of CC cases attributable to HR‐HPV infection [3–5]. HR‐HPV induces malignant transformation by promoting uncontrolled cellular proliferation, genomic instability and, importantly, by modulating host antiviral and immune responses to evade immune surveillance [6, 7]. In contrast, HPV‐negative CC represents a rare subset, accounting for only 2%–8% of cases [8, 9].

Emerging evidence suggests that immune escape is a hallmark of HPV‐positive CC, driven by the virus’s ability to alter the tumor immune microenvironment [10]. HR‐HPV oncoproteins, such as E6 and E7, suppress innate immune signaling, disrupt interferon pathways, and impair antigen processing and presentation, thereby reducing antitumor immune activation [11, 12]. Additionally, HPV‐positive tumors exhibit unique immune infiltration patterns characterized by altered dendritic cell function, exhausted T‐cell phenotypes, and dysregulation of cytokine and chemokine networks [13–15]. Collectively, these changes create an immune‐permissive environment that facilitates persistent viral infection and tumor progression [16, 17].

Despite these findings, the specific molecular determinants and regulatory pathways governing immune dysregulation in HPV‐positive CC remain inadequately defined [18, 19]. In particular, the key genes linking HPV‐associated oncogenic signaling to immune modulation—and their expression differences between HPV‐positive and HPV‐negative tumors—require further elucidation [20, 21]. A deeper understanding of these immune‐related molecular features is essential for identifying novel biomarkers and therapeutic targets specific to HPV‐associated CC [22, 23].

This study integrated bulk RNA‐sequencing data from The Cancer Genome Atlas (TCGA)–CESC with single‐cell RNA‐sequencing datasets from Gene Expression Omnibus (GEO) to explore molecular alterations associated with HPV infection. By employing gene set enrichment analysis (GSEA), multiple machine learning algorithms, and comprehensive immune infiltration profiling, this study aims to discover previously unrecognized immune‐evasion signature genes and their regulatory networks in HPV‐associated CC. Key molecular targets were validated in CC cell lines, with a particular focus on investigating the functional role of IFNGR1 in the progression of HPV‐positive disease. Systematically mapping these HPV‐associated immune regulatory mechanisms reveals molecular features that have yet to be fully characterized and highlights potential targets that may pave the way for mechanistic research and therapeutic innovation in HPV‐associated CC.

## 2. Materials and Methods

### 2.1. Data Sources

The datasets utilized in this study were sourced from two public repositories: the GEO (https://www.ncbi.nlm.nih.gov/geo/) [24] and TCGA [25]. The TCGA‐CESC cohort comprises high‐throughput sequencing data from a total of 309 CC specimens, including 304 primary tumor samples, 11 adjacent normal tissues, and 6 recurrent tumors. The HPV infection status was documented for 178 of the 304 tumor samples, which included 169 HPV‐positive and 9 HPV‐negative cases. Only samples with recorded HPV status were included in subsequent analyses.

The single‐cell RNA sequencing dataset GSE171894, generated using the Illumina HiSeq 2500 platform (*Homo sapiens*), encompasses four CC samples: two HPV‐positive and two HPV‐negative cases. All samples were incorporated into downstream analyses. The GSE197461 dataset, sequenced on the Illumina NovaSeq 6000 platform (*H. sapiens*), comprises eight CC samples, with six classified as HPV‐positive and two as HPV‐negative; all samples were included in the subsequent statistical analyses.

### 2.2. Differential Expression Analysis and GSEA Enrichment Analysis

Differential expression analysis for the TCGA‐CESC dataset was conducted using the DESeq2 (v1.46.0) R package [26]. Genes were considered significantly differentially expressed between HPV‐positive and HPV‐negative groups if they exhibited an adjusted *p*‐value  < 0.05 and |log_2_ fold change (FC)| > 1.

GSEA was performed to determine whether predefined gene sets displayed consistent expression differences between the two biological states. All genes were ranked according to differential expression statistics, where the *p*‐value indicates the significance of expression differences and log_2_FC denotes the magnitude of fold change (log_2_‐transformed). Gene symbols served as gene identifiers. Enrichment analysis utilized the GSEA function in the clusterProfiler package (v4.14.6) [27], with parameters set as eps = 0, minGSSize = 10, and maxGSSize = 1000; all other parameters remained at default values. The top 15 enriched pathways in the HPV‐positive and HPV‐negative CC groups were selected based on the *p*‐value and normalized enrichment score (NES). Visualization of enrichment results was conducted using the GseaVis package (v0.1.0).

### 2.3. Gene Ontology (GO) and Kyoto Encyclopedia of Genes and Genomes (KEGG Enrichment Analyses)

GO and the KEGG comprise predefined gene sets categorized by specific biological functions or pathways. GO and KEGG enrichment analyses employed hypergeometric testing to assess whether genes within these predefined sets appeared at significantly different frequencies among the differentially expressed genes (DEGs) compared to the background gene set. In this study, GO and KEGG enrichment analyses were performed on the intersecting genes between DEGs from HPV‐positive and HPV‐negative CC samples and immune escape‐related gene sets using the clusterProfiler R package (v4.14.6) [27]. These analyses aimed to elucidate the major biological functions associated with the differentially expressed immune escape‐related genes.

### 2.4. Machine Learning‐Based Identification of Feature Genes

To identify immune escape‐related DEGs most strongly associated with HPV infection status, three machine learning algorithms were employed: LASSO regression, Random Forest, and SVM‐RFE [28, 29]. LASSO regression utilizes an L1 regularization term that facilitates automatic feature selection by shrinking certain coefficients to zero. This approach contrasts with L2 regularization (ridge regression), which reduces coefficient magnitudes but does not eliminate less informative features. As the regularization parameter λ increases, penalization intensifies, leading to a greater number of coefficients being reduced to zero, thereby enhancing feature selection. The optimal λ was determined using 10‐fold cross‐validation with the “lambda.min” criterion via the glmnet package (v4.1‐8). Random Forest, an ensemble learning method, constructs multiple decision trees and aggregates their predictions to improve accuracy and robustness. In addition to its predictive strength, Random Forest is widely utilized for feature selection, providing quantitative assessments of each feature’s importance in model performance. In this study, 500 trees were generated (ntree = 500), and the number of features considered at each split was set to the square root of the total number of input features (mtry = sqrt(p)), implemented through the randomForest package (v4.7‐1.1). The SVM‐RFE algorithm iteratively trains a support vector machine while removing the least informative features at each step based on feature importance rankings. This recursive elimination strategy minimizes the impact of the removed features on the model, resulting in an optimized subset of predictive variables. A prominent advantage of SVM‐RFE is its enhancement of model generalizability while automating feature selection without extensive manual parameter tuning. SVM‐RFE was executed using a linear kernel with a cost parameter *C* = 1, implemented via the e1071 package (v1.7‐14), with 10‐fold cross‐validation employed to evaluate feature subsets. The intersection of gene sets identified by all three algorithms constituted the final set of HPV infection‐related characteristic genes in CC.

### 2.5. Transcriptional Regulatory Analysis

To investigate the transcriptional regulatory networks of the three target genes, transcription factors and their corresponding regulatory relationships were retrieved from the TRRUST database (https://www.grnpedia.org/trrust/). Transcription factors specifically regulating the three key genes were extracted to construct their regulatory networks, with visualization performed using the ggraph package (v2.2.1).

### 2.6. miRNA Regulatory Analysis

StarBase (ENCORI, https://rnasysu.com/encori/) serves as an integrated RNA interaction analysis platform that combines high‐throughput sequencing data, including CLIP‐seq and degradome‐seq, to provide comprehensive analyses of regulatory networks involving miRNAs, degradome RNAs, and RNA‐binding proteins (RBPs). In this study, noncoding RNAs capable of targeting the key genes were extracted to investigate potential regulatory influences on their expression. The resulting miRNA–gene regulatory networks were visualized utilizing the ggraph package (v2.2.1).

### 2.7. Immune Infiltration Analysis

Tumor tissues are typically infiltrated by a diverse array of immune cells that interact within complex networks. Immune infiltration analysis examines the proportional composition of various immune cell types within the tissue. This study utilized the CIBERSORT algorithm to estimate the relative abundance of immune cells in transcriptomic samples. CIBERSORT (v0.1.0) employs linear support vector regression to deconvolute gene expression matrices, offering estimates for the proportions of 22 immune cell types based on a reference training dataset. To assess differences in immune cell infiltration between subtypes, rank‐sum tests (Wilcoxon test) were conducted on the infiltration levels of each immune cell type, enabling the identification of immune cell populations that exhibited significant differences between HPV‐positive and HPV‐negative CC groups.

### 2.8. Single‐Cell Data Analysis

The GSE171894 single‐cell RNA sequencing dataset of CC served for single‐cell analyses in this study. Raw single‐cell data were imported into the R environment utilizing the DropletUtils package (v1.24.0) [30]. Low‐quality cells were filtered based on sequencing depth (total counts), the number of detected genes, and the proportion of UMI counts mapped to mitochondrial genes (mitochondrial percentage). Specifically, cells with fewer than 200 detected genes, more than 6000 detected genes, or a mitochondrial read proportion exceeding 20% were excluded as low‐quality or potentially doublet cells. Subsequent downstream analyses employed the following R packages: scran (v1.34.0) [31] for normalization and identification of highly variable genes; batchelor (v1.22.0) [32] for batch effect correction; bluster (v1.16.0) [33] for clustering; and scater (v1.34.1) [34] for dimensional reduction and quality control. Cell type annotation relied on known marker genes.

### 2.9. Tumor Cell Identification

The inferCNV (v1.22.0) R package is specifically designed for single‐cell RNA sequencing data to infer copy number variations (CNVs) and identify malignant tumor cells within samples. This method estimates large‐scale chromosomal amplifications or deletions by comparing gene expression profiles between target cells and reference cells. In this study, immune cells served as reference cells to determine which epithelial cell clusters in HPV‐positive and HPV‐negative samples corresponded to tumor cells.

### 2.10. Single‐sample GSEA (ssGSEA)

ssGSEA extends traditional GSEA to facilitate gene set enrichment scoring at the individual sample level, providing a more granular assessment of pathway activity across heterogeneous samples. ssGSEA applies rank‐based normalization to gene expression data, wherein genes in each sample are ranked by expression level, and these rank values generate an empirical cumulative distribution function (ECDF) for each gene set. This rank‐based method minimizes the influence of absolute expression differences, enhancing the comparability of enrichment scores across samples. In this study, the GSVA R package (v2.0.7) [35] was employed to calculate enrichment scores of CC‐related signature genes across all single cells. Differences in enrichment scores between HPV‐positive and HPV‐negative cells were subsequently evaluated for each cell type to identify populations most impacted by HPV infection.

### 2.11. Survival Analysis

To evaluate the clinical relevance of the three signature genes in HPV‐positive CC, Kaplan–Meier survival analysis was performed for overall survival (OS) using the TCGA‐CESC cohort. Only patients with HPV‐positive tumors and non‐missing follow‐up data were included. OS time was defined as days from diagnosis to death or last follow‐up, converted to months. Patients were stratified into high‐ and low‐expression groups according to the median normalized expression of each signature gene (IFNGR1, TRADD, and PSMB9). Kaplan–Meier survival curves were generated with the survival and survminer R packages, and group differences were assessed using the log‐rank test. Hazard ratios (HRs) and 95% confidence intervals (CIs) were estimated by univariate Cox proportional hazards regression. A two‐sided *p*‐value  < 0.05 was considered statistically significant.

### 2.12. Cell Culture and Transfection

HPV‐positive CC cell lines HeLa (HPV18+, RRID: CVCL_0030) and SiHa (HPV16+, RRID: CVCL_0032), along with the HPV‐negative CC cell line C33A (RRID: CVCL_1094) and normal human keratinocyte cell line W12 (RRID: CVCL_T290), were acquired from Tongpai Biotechnology Co., Ltd. (Shanghai, China). Cells were cultured in DMEM supplemented with 10% fetal bovine serum (FBS) and 1% penicillin‐streptomycin under standard conditions (37°C, 5% CO_2_). For knockdown experiments, HeLa and SiHa cells were transfected with siRNAs targeting IFNGR1 (si‐IFNGR1#1 and si‐IFNGR1#2) or a negative control siRNA (si‐NC) using Lipofectamine 3000 (Thermo Fisher Scientific, USA) as per the manufacturer’s instructions. si‐IFNGR1#1, si‐IFNGR1#2, and si‐NC were chemically synthesized by GenePharma Co., Ltd. (Shanghai, China) with high‐performance liquid chromatography purification (purity ≥ 95%). Transfection efficiency was confirmed via real‐time quantitative polymerase chain reaction (RT‐qPCR) and Western blot analysis 48 h post‐transfection.

### 2.13. RNA Extraction and RT‐qPCR

Total RNA was extracted from cells using the TRIzol reagent (Invitrogen, USA). RNA concentration and purity were measured with a NanoDrop 2000 spectrophotometer (Thermo Fisher Scientific, USA). Complementary DNA (cDNA) synthesis was conducted using the PrimeScript RT Reagent Kit (Takara, Japan). RT‐qPCR was performed on a QuantStudio 6 Flex Real‐Time PCR System (Applied Biosystems, USA) using the SYBR Green Master Mix (Thermo Fisher Scientific, USA). Relative gene expression levels of IFNGR1, TRADD, and PSMB9 were calculated using the 2^−ΔΔCt^ method and normalized to GAPDH. The primer sequences utilized for quantitative RT‐qPCR analysis are detailed in Table 1.

**Table 1:** Primer sequences used for qPCR analysis.

Gene	Forward primer (5′‐3′)	Reverse primer (5′‐3′)
IFNGR1	TTGAACTCGTCGGTTGCCTT	AAACAAGCGGCGTGATTTGT
TRADD	GGAAGCGGCGGAGTAGAG	TCTCACCTCCTGCTGCACTA
PSMB9	TGCAGCATATAAGCCAGGCA	ACCATACCAGGTTTTGGCCC
GAPDH	CGGAGTCAACGGATTTGGTCGTAT	AGCCTTCTCCATGGTGGTGAAGAC

### 2.14. Western Blot Analysis

Total protein was extracted from cells using RIPA buffer (Beyotime, China) supplemented with protease inhibitors. Protein concentrations were assessed via the BCA assay (Pierce, USA). Equal amounts of protein were separated by SDS‐PAGE and transferred onto PVDF membranes (Millipore, USA). Membranes were blocked with 5% non‐fat milk for 1 h and incubated overnight at 4°C with primary antibodies against IFNGR1 (1:1000, #AF7734, Affinity), TRADD (1:1000, #DF6279, Affinity), and PSMB9 (1:1000, #DF6606, Affinity). For internal normalization, GAPDH (1:5000, AB0037, Abways) was used as the loading control for IFNGR1 and PSMB9 blots, while β‐actin (1:5000, AB8227, Abways) served as the loading control specifically for TRADD immunoblotting, based on stable expression validation in cervical cell lines used in this study. Following washing, membranes were incubated with HRP‐conjugated secondary antibodies, goat anti‐rabbit IgG(H + L)HRP (1:5000, #S0001, Affinity), for 1 h at room temperature. Protein bands were visualized using an enhanced chemiluminescence kit (ECL, Thermo Fisher Scientific, USA) and quantified using ImageJ software.

### 2.15. Cell Proliferation Assays

Cell viability was assessed using the Cell Counting Kit‐8 (CCK‐8) assay (Dojindo, Japan). Briefly, transfected HeLa and SiHa cells were seeded in 96‐well plates (3 × 10^3^ cells/well) and incubated for 24, 48, and 72 h. CCK‐8 solution was added to each well and incubated for 2 h at 37°C. Absorbance at 450 nm was measured using a microplate reader (Bio‐Rad, USA). Colony formation assays involved seeding 500 transfected cells in 6‐well plates, followed by culture for 10–14 days. Colonies were fixed with 4% paraformaldehyde (Beyotime, China), stained with 0.1% crystal violet (Sigma–Aldrich, USA), and counted manually.

### 2.16. Migration and Invasion Assays

Transwell assays were performed using 24‐well Transwell chambers (8 μm pore size, Corning, USA). In migration assays, 5 × 10^4^ transfected cells in serum‐free medium were placed in the upper chamber, while a medium containing 10% FBS was added to the lower chamber. In invasion assays, the upper chambers were precoated with Matrigel (BD Biosciences, USA). After 24 h, cells on the lower surface were fixed, stained with crystal violet, and counted under a microscope.

### 2.17. Apoptosis Analysis

Apoptosis was evaluated by flow cytometry with the Annexin V‐FITC/PI apoptosis detection kit (BD Biosciences, USA). Transfected HeLa and SiHa cells were harvested 48 h post‐transfection, washed with PBS, and stained according to the manufacturer’s protocol. Quantification of apoptotic cells was performed using a FACSCalibur flow cytometer (BD Biosciences, USA) and analyzed using FlowJo software (BD Biosciences, USA).

### 2.18. Statistical Analysis

All analyses were performed using R software (v4.4.1) and GraphPad Prism 9.0 (GraphPad Software, San Diego, California, USA). A *p*‐value  < 0.05 was considered statistically significant. For continuous variables, Student’s *t*‐test was applied to normally distributed data, while the Wilcoxon rank‐sum test was utilized for non‐normally distributed data. Categorical variables were compared using the chi‐square test.

## 3. Results

### 3.1. Differential Expression Analysis Between HPV‐Positive and HPV‐Negative CC

**Figure 1:**
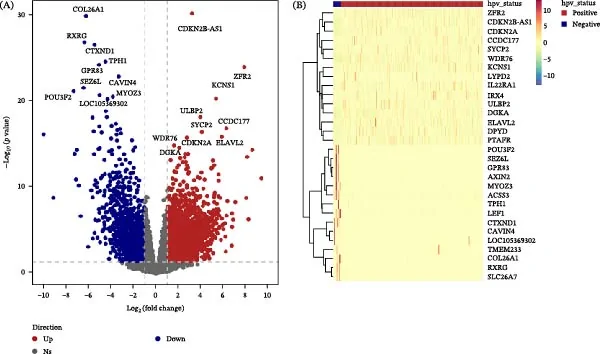
Differential gene expression analysis between HPV‐positive and HPV‐negative cervical cancers. (A) Volcano plot displaying 4266 DEGs. (B) Heatmap showing the top 15 upregulated and top 15 downregulated genes in each group.

To investigate the functional impact of HPV infection on CC, differential expression analysis was performed between HPV‐positive and HPV‐negative tumors. Using thresholds for the *p*‐value and log_2_FC, a total of 4266 DEGs were identified, including 2635 upregulated and 1631 downregulated genes in HPV‐positive CC (Supporting Information 1: Table S1). The overall distribution of DEGs is depicted through a volcano plot (Figure 1A), while a heatmap illustrates the expression patterns of the top 15 upregulated and top 15 downregulated genes in HPV‐positive tumors compared with HPV‐negative tumors (Figure 1B).

### 3.2. Functional Enrichment Analysis Between HPV‐Positive and HPV‐Negative Groups

To characterize the functional alterations associated with HPV infection, GSEA was conducted using KEGG pathway gene sets to compare HPV‐positive and HPV‐negative CC samples. Pathways related to viral infection, such as herpes simplex virus 1 infection, Influenza A, and viral protein interaction with cytokines and cytokine receptors, as well as immune‐related pathways including IL‐17 signaling, natural killer cell‐mediated cytotoxicity, NOD‐like receptor signaling, and antigen processing and presentation, were significantly enriched in the HPV‐positive group. Conversely, classical tumor‐associated pathways, including Wnt signaling, Hedgehog signaling, and Rap1 signaling, were predominantly enriched in HPV‐negative tumors (Figure 2A). The top five significantly enriched pathways in both HPV‐positive and HPV‐negative groups, alongside their corresponding enrichment statistics, were visualized (Figure 2B,C).

Figure 2GSEA functional enrichment analysis. (A) Top 15 pathways enriched in HPV‐positive (red) and HPV‐negative cervical cancer samples. (B) Top 5 significantly enriched pathways in HPV‐positive tumors. (C) Top 5 significantly enriched pathways in HPV‐negative tumors.

**Figure:**
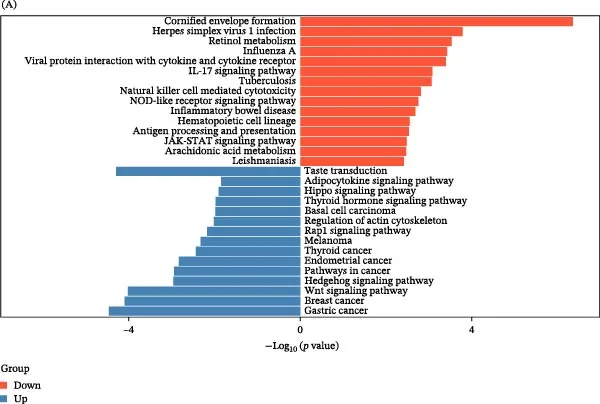

**Figure:**
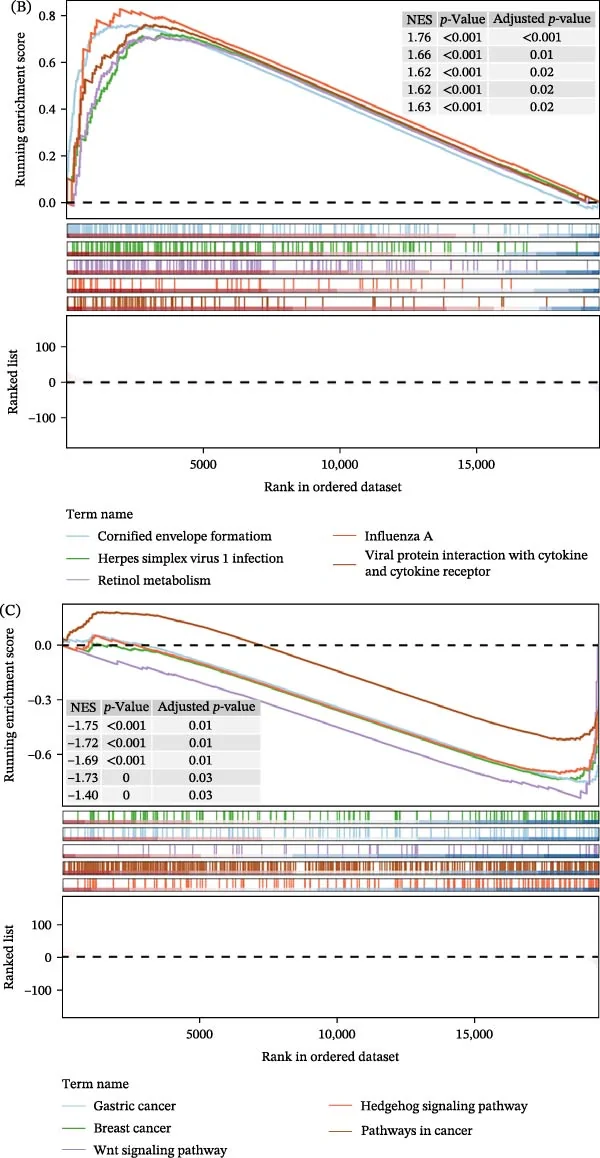

### 3.3. Machine Learning‐Based Identification of HPV‐Related Immune Evasion Signature Genes in CC

A previous study employing a genome‐wide CRISPR screen identified 182 genes associated with tumor immune evasion in murine cancer cell lines [36]. To assess their relevance in CC, these murine genes were converted to human orthologs, resulting in 180 corresponding genes (Supporting Information 2: Table S2). By intersecting these genes with the DEGs between HPV‐positive and HPV‐negative tumors, 11 immune evasion‐related genes were identified as dysregulated between the two groups (Figure 3A), including B2M, HLA‐A, IFNGR1, IPPK, IRF1, PSMB9, STAT1, TAP1, TAP2, TNFAIP3, and TRADD. Three machine learning algorithms—LASSO regression (Figure 3B,C), Random Forest (Figure 3D), and SVM‐RFE (Figure 3E,F)—were subsequently applied to select HPV‐associated immune evasion signature genes from the 11 candidates. Three robust overlapping signature genes consistently screened out by all three algorithms were IFNGR1, TRADD, and PSMB9. The overlapping genes identified by all three algorithms were regarded as robust HPV‐related signature genes (Figure 3G). The expression patterns of these signature genes (IFNGR1, TRADD, and PSMB9) in HPV‐positive and HPV‐negative CC samples were then assessed (Figure 3H).

Figure 3Machine learning selection of HPV‐associated immune‐evasion feature genes. (A) Venn diagram depicting the intersection between DEGs and the immune‐evasion gene set. (B) LASSO regression path for each variable. (C) LASSO cross‐validation plot. (D) Number of trees in the random forest and three types of error curves. (E) SVM‐RFE feature number versus error curve. (F) SVM‐RFE feature count–accuracy curve. (G) Intersection of selected genes. (H) Expression levels of feature genes between HPV‐positive and HPV‐negative groups.

**Figure:**
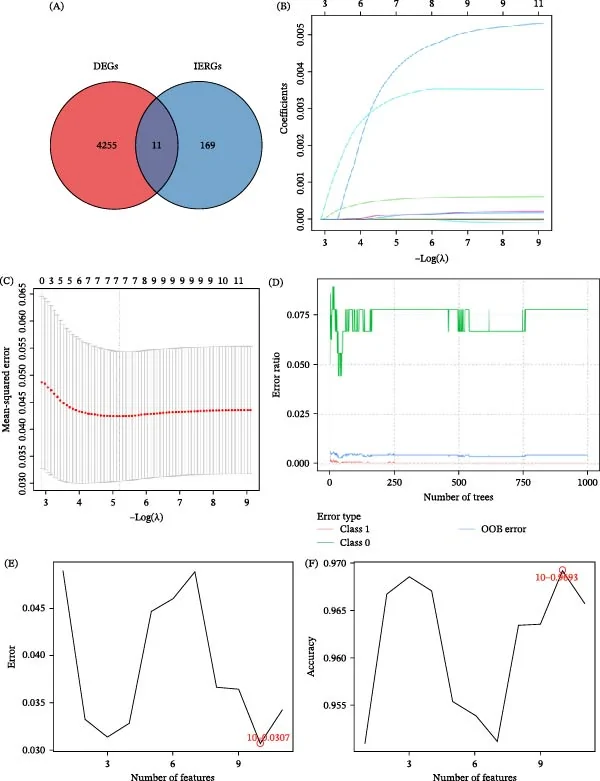

**Figure:**
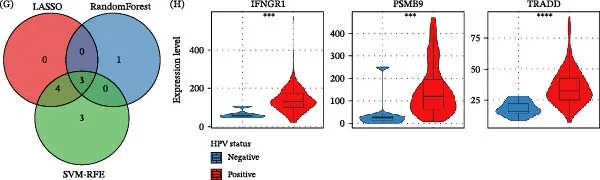

### 3.4. GO and KEGG Enrichment Analyses of Signature Genes

**Figure 4:**
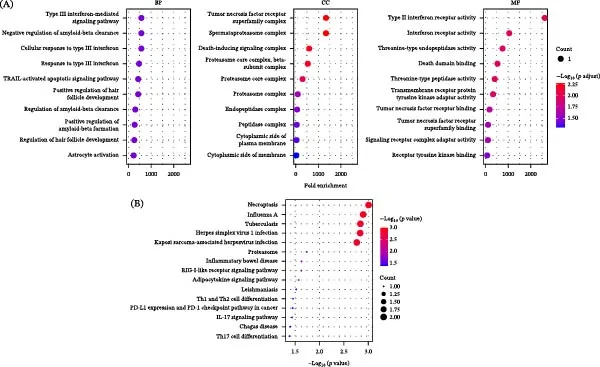
GO and KEGG enrichment analyses of feature genes. (A) GO enrichment analysis showing the top 10 significantly enriched terms in each category. (B) KEGG enrichment analysis displaying the top 15 pathways enriched among feature genes.

To elucidate the biological functions associated with the identified signature genes, both GO and KEGG enrichment analyses were performed. GO enrichment analysis revealed significant enrichment in multiple interferon‐related gene sets, including the type III interferon‐mediated signaling pathway (BP), tumor necrosis factor receptor superfamily complex (CC), and type II interferon receptor activity (MF) (Figure 4A). Interferons play a critical role as cellular defense mechanisms against viral infection. KEGG pathway analysis further demonstrated enrichment of the signature genes in the necroptosis pathway, a regulated form of inflammatory cell death, as well as in various viral infection‐related pathways such as Influenza A, Tuberculosis, and herpes simplex virus 1 infection (Figure 4B). Collectively, these findings suggest that HPV‐associated immune evasion genes in CC are strongly linked to interferon signaling, antiviral responses, and programmed inflammatory cell death.

### 3.5. Regulatory Network of Signature Genes

**Figure 5:**
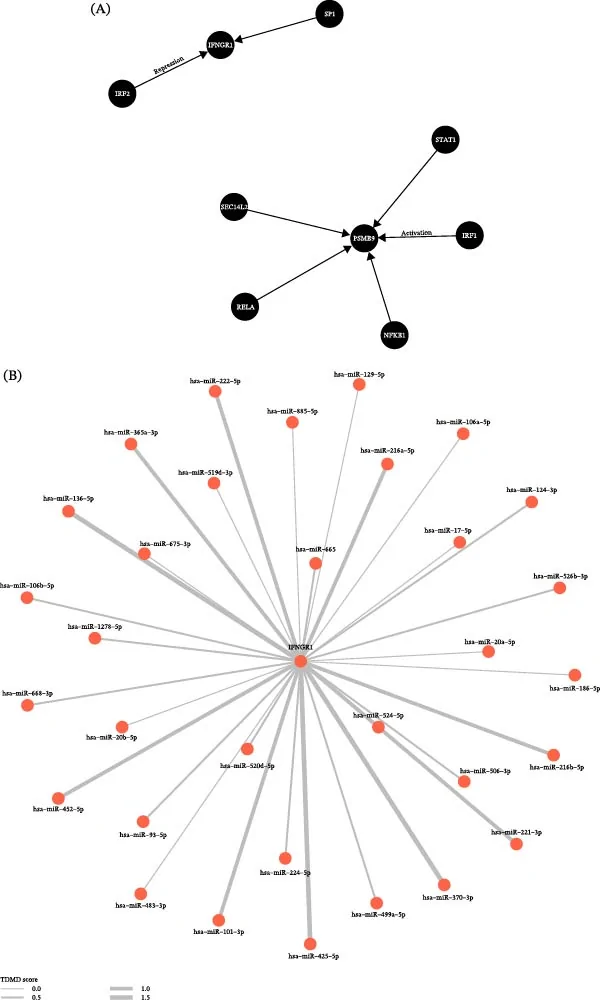
Regulatory network of feature genes. (A) Transcription factor–feature gene regulatory network. (B) miRNA–feature gene regulatory network.

To elucidate the upstream regulatory mechanisms of the signature genes, potential transcription factors controlling their expression were analyzed. Results indicated that IRF2 may repress IFNGR1 transcription, while IRF1 appears to positively regulate PSMB9 expression (Figure 5A). Additionally, miRNA regulatory network analysis suggested that hsa‐miR‐370‐3p, hsa‐miR‐425‐5p, and hsa‐miR‐136‐5p likely target IFNGR1 for degradation, thereby modulating its expression levels (Figure 5B).

### 3.6. Immune Infiltration Analysis

To investigate the immune cell landscape in HPV‐positive and HPV‐negative CC samples, immune infiltration analysis was performed across all samples (Figure 6A). The analysis identified notable populations of macrophage M0, activated dendritic cells, plasma cells, and resting dendritic cells. Among these, activated dendritic cells, plasma cells, and resting dendritic cells exhibited significantly higher infiltration levels in the HPV‐positive group compared to the HPV‐negative group, while macrophage M0 infiltration was significantly elevated in the HPV‐negative group (Figure 6B). Furthermore, correlation analysis revealed a significant positive correlation between TRADD expression and the infiltration level of activated dendritic cells (Figure 6C), suggesting a potential role of TRADD in modulating the immune microenvironment in HPV‐positive CC.

Figure 6Immune infiltration analysis. (A) Proportional composition of immune cell types in each sample from HPV‐positive and HPV‐negative groups. (B) Boxplots showing differences in immune cell infiltration levels between HPV‐positive and HPV‐negative samples. (C) Correlation analysis between feature gene expression and immune cell infiltration levels.

**Figure:**
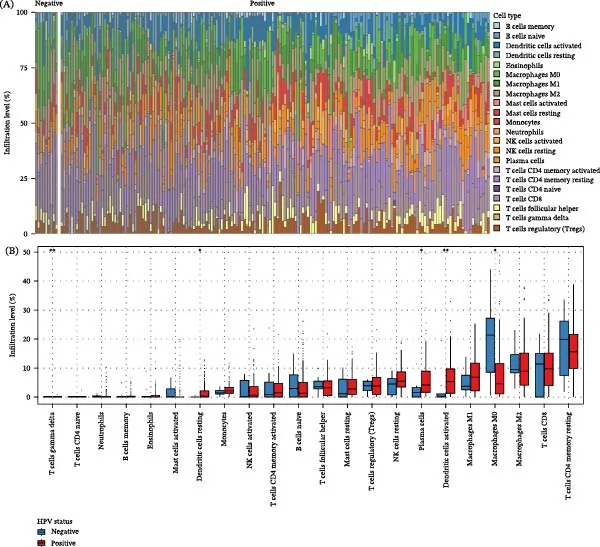

**Figure:**
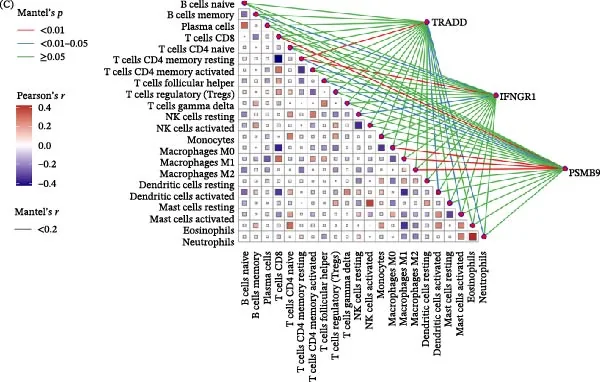

### 3.7. Single‐Cell Atlas of HPV‐Positive and HPV‐Negative CC and Tumor Cell Identification

**Figure 7:**
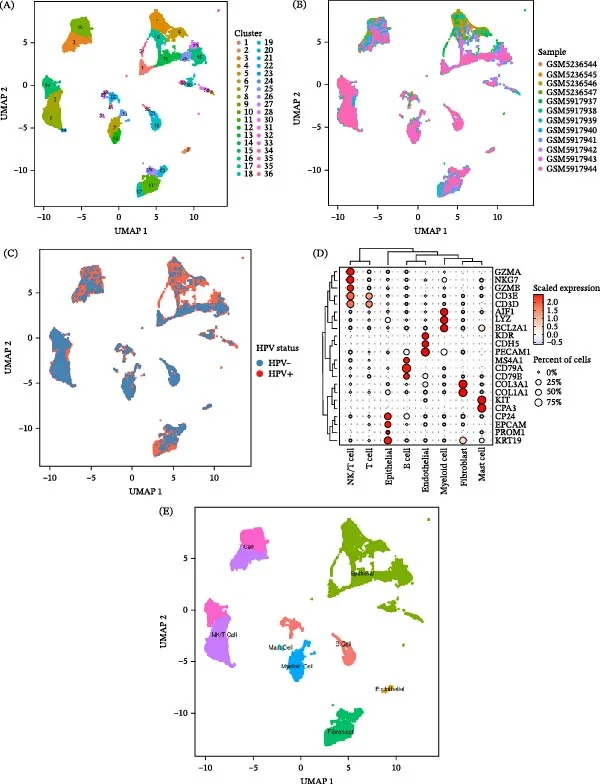
Construction of single‐cell atlas of cervical cancer. (A) Unsupervised clustering of all cells. (B) Sample origin of each cell. (C) HPV infection status of cells from different samples. (D) Marker genes used for cell type annotation. (E) Identification of all cell types using marker genes.

**Figure 8:**
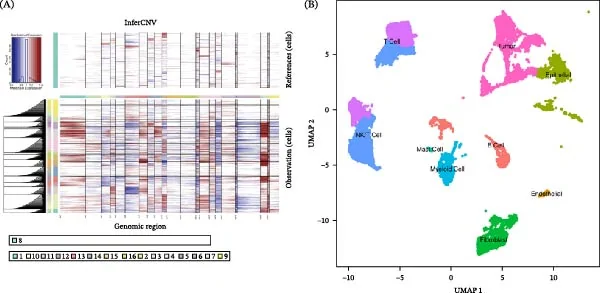
Tumor cell identification. (A) Copy number variation analysis of each cell relative to reference cluster 8. (B) UMAP visualization of all cells following tumor cell identification.

To explore the relationship between signature genes and cellular composition in CC tissues, a single‐cell atlas of CC was constructed (Figure 7A–E). Following quality control, a total of 49,560 high‐quality cells were retained, including 35,368 from the HPV‐positive group and 14,192 from the HPV‐negative group. Given that CC is an epithelial‐derived malignancy, CNV analysis was applied to distinguish epithelial cells likely representing malignant tumor cells (Figure 8A). Based on this analysis, clusters 1, 4, 6, 13, 16, and 34 were identified as probable tumor cell populations (Figure 8B), establishing a foundation for subsequent exploration of signature gene expression within malignant and non‐malignant cell types.

### 3.8. Identification of Cell Types Enriched for Feature Gene Expression

**Figure 9:**
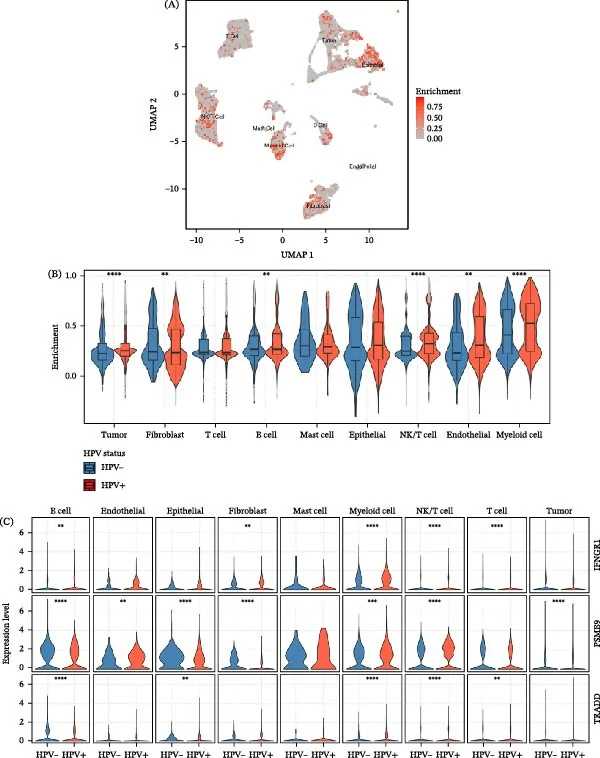
Identification of cells expressing HPV‐associated feature genes. (A) UMAP plot showing ssGSEA enrichment scores of feature genes across all cells. (B) Statistical comparison of enrichment scores across cell types between HPV‐positive and HPV‐negative samples. (C) Expression levels of feature genes across all cell types.

To determine which cell types predominantly express the signature genes, ssGSEA was performed using the signature genes as the gene set. Enrichment scores across all cells were visualized using a UMAP plot (Figure 9A). The results demonstrated that signature genes are highly expressed in epithelial cells and myeloid cells. Statistical analysis revealed significant differences in enrichment scores among myeloid cells, endothelial cells, tumor cells, lymphocytes, and fibroblasts between HPV‐positive and HPV‐negative tissues, with enrichment scores markedly higher in the HPV‐positive group (Figure 9B). Finally, expression levels of the three signature genes across all cell types were examined (Figure 9C), revealing that IFNGR1 and TRADD exhibited the greatest expression differences in immune cells, while PSMB9 displayed the most significant differences in both immune cells and tumor cells.

### 3.9. OS Analysis of Signature Genes in HPV‐Positive CC

**Figure 10:**
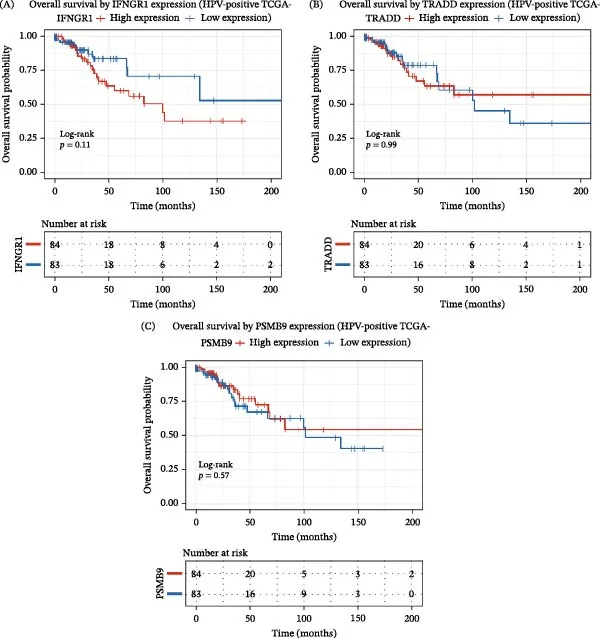
Kaplan–Meier analysis of overall survival in HPV‐positive cervical cancer patients stratified by signature gene expression. (A) Overall survival by IFNGR1 expression. (B) Overall survival by TRADD expression. (C) Overall survival by PSMB9 expression. Patients were divided into high‐ and low‐expression groups using the median normalized expression as the cutoff. *p* values were calculated by log‐rank test; hazard ratios (HRs) and 95% confidence intervals (CIs) were estimated by univariate Cox regression.

We further assessed whether the expression levels of IFNGR1, TRADD, and PSMB9 were associated with clinical outcomes in the HPV‐positive TCGA‐CESC cohort (*n* = 167 with valid OS data and 33 death events). Kaplan–Meier analysis showed that patients with high IFNGR1 expression had a consistent trend toward shorter OS compared with those with low IFNGR1 expression, although this did not reach statistical significance (HR = 1.78, 95% CI: 0.86–3.68; log‐rank *p* = 0.113; Figure 10A). In contrast, TRADD and PSMB9 expression levels were not associated with OS in this cohort (TRADD: HR = 1.01, 95% CI: 0.51–1.99; log‐rank *p* = 0.988; PSMB9: HR = 0.82, 95% CI: 0.41–1.63; log‐rank *p* = 0.571; Figure 10B,C). These findings suggest that, among the three immune‐evasion signature genes, IFNGR1 may have the strongest clinical relevance in HPV‐positive CC, corroborating its functional role in promoting tumor progression.

### 3.10. IFNGR1 Is Highly Expressed in HPV‐Positive CC Cells

**Figure 11:**
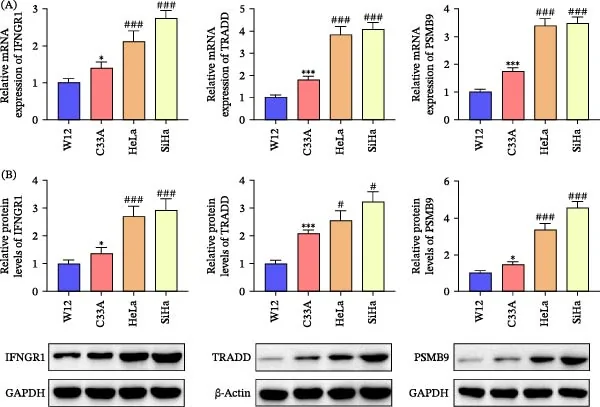
IFNGR1 is highly expressed in HPV‐positive cervical cancer cells. (A) RT‐qPCR analysis of IFNGR1, TRADD, and PSMB9 mRNA expression in HPV18+ (HeLa), HPV16+ (SiHa), HPV‐negative (C33A) cervical cancer cell lines, and normal keratinocytes (W12). (B) Western blot analysis of IFNGR1, TRADD, and PSMB9 protein levels in the same cell lines.  ^∗^indicates a significant difference compared to the W12 group (*p* < 0.05) and  ^∗∗∗^(*p* < 0.001); ^#^ indicates a significant difference compared to the C33A group (*p* < 0.05), ^##^(*p* < 0.01), and ^###^(*p* < 0.001).

To validate the expression of the signature genes in CC cell lines, both mRNA and protein levels of IFNGR1, TRADD, and PSMB9 were measured across different cell line groups, including two CC cell lines, HeLa (HPV18+) and SiHa (HPV16+), one HPV‐negative CC cell line (C33A), and one normal human keratinocyte cell line (W12). RT‐qPCR results indicated that IFNGR1 expression was highest in HPV‐positive HeLa and SiHa cells, followed by HPV‐negative C33A cells, with the lowest levels observed in W12 cells. TRADD mRNA levels exhibited a similar pattern, where C33A cells showed significantly higher expression than W12, and HeLa and SiHa cells had the highest expression across all groups. In contrast, PSMB9 expression was significantly elevated in HPV‐positive HeLa and SiHa cells compared to C33A and W12 cells, with no notable difference between C33A and W12 (Figure 11A). Consistent with these findings, Western blot analysis confirmed that protein levels of IFNGR1 and TRADD were substantially increased in HeLa and SiHa cells relative to both C33A and W12, while C33A also exhibited higher levels than W12. PSMB9 protein expression was markedly elevated in HeLa and SiHa cells compared to C33A and W12, corroborating the mRNA data (Figure 11B). Collectively, these results indicate varying degrees of upregulation of the three signature genes in CC cells, with the highest expression consistently observed in HPV‐positive cell lines. This graded expression pattern suggests that HPV infection may enhance the expression of these genes beyond levels seen in HPV‐negative cancer cells.

### 3.11. Knockdown of IFNGR1 Suppresses Biological Functions of HPV‐Positive CC Cells

To investigate the functional role of IFNGR1 in HPV‐positive CC, HeLa and SiHa cells were transfected with siRNAs targeting IFNGR1 (si‐IFNGR1#1 and si‐IFNGR1#2) or a si‐NC. RT‐qPCR and Western blot analyses confirmed that both siRNAs effectively reduced IFNGR1 mRNA and protein expression compared to si‐NC (Figure 12A,B). Among these, si‐IFNGR1#1 exhibited a superior knockdown efficiency and was therefore selected for subsequent functional assays. Cell proliferation assays demonstrated that IFNGR1 knockdown significantly suppressed the growth of HPV‐positive CC cells (HeLa and SiHa). CCK‐8 assays revealed a notable reduction in cell viability over time, while colony formation assays indicated fewer and smaller colonies in IFNGR1‐silenced cells compared to controls (Figure 12C,D). Migration and invasion assays performed using Transwell chambers indicated that silencing IFNGR1 substantially decreased both the migratory and invasive capacities of HeLa and SiHa cells (Figure 12E,F). Flow cytometry analysis further revealed that IFNGR1 knockdown significantly increased apoptosis in HPV‐positive CC cells relative to si‐NC controls (Figure 12G). Collectively, these findings suggest that IFNGR1 promotes proliferation, migration, and invasion while inhibiting apoptosis in HPV‐positive CC cells, emphasizing its role as a key functional regulator and potential therapeutic target in HPV‐associated CC.

Figure 12IFNGR1 knockdown inhibits the biological behavior of HPV‐positive cervical cancer cells. (A) RT‐qPCR analysis of IFNGR1 expression in HeLa and SiHa cells transfected with si‐NC, si‐IFNGR1#1, or si‐IFNGR1#2. (B) Western blot analysis of IFNGR1 protein levels in transfected cells. (C) CCK‐8 and (D) colony formation assays showing the effects of IFNGR1 knockdown on cell proliferation. (E–F) Transwell assays demonstrating the effects of IFNGR1 knockdown on migration (E) and invasion (F). (G) Flow cytometry analysis of apoptosis in HPV‐positive cervical cancer cells following IFNGR1 knockdown.  ^∗^indicates a significant difference compared to the si‐NC group (*p* < 0.05);  ^∗∗^(*p* < 0.01), and  ^∗∗∗^(*p* < 0.001).

**Figure:**
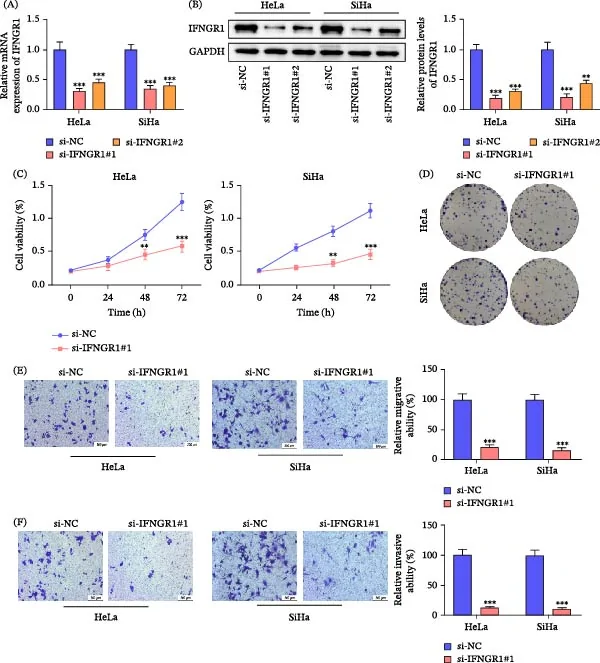

**Figure:**
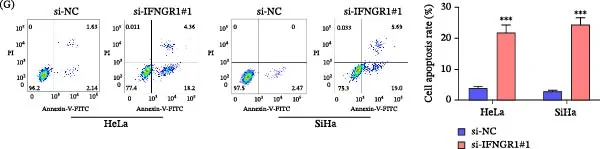

## 4. Discussion

In this study, a systematic investigation of the molecular and cellular differences between HPV‐positive and HPV‐negative CC was conducted. A total of 6266 DEGs were identified, with significant enrichment in immune‐related pathways, particularly interferon signaling and viral response pathways, observed in HPV‐positive tumors. Utilizing a combination of LASSO, Random Forest, and SVM‐RFE algorithms, IFNGR1, TRADD, and PSMB9 emerged as key signature genes linked to HPV‐associated immune evasion. Single‐cell transcriptomic analysis revealed predominant expression of these genes in epithelial and myeloid cell populations, with expression correlating to immune cell infiltration. Functional experiments demonstrated that silencing HPV‐associated immune regulators (IFNGR1) significantly suppressed proliferation, migration, and invasion while promoting apoptosis in HPV‐positive CC cells, highlighting their potential oncogenic roles.

These findings offer new insights into how HPV reshapes the immune microenvironment to facilitate tumor persistence. In line with existing knowledge that HR‐HPV manipulates host antiviral defenses [22, 37, 38], significant enrichment of interferon‐mediated pathways and antigen presentation machinery among HPV‐associated genes was observed. Additionally, remodeling of the immune landscape is often accompanied by metabolic shifts within the tumor microenvironment. Recent studies have indicated that arginine metabolism can drive the polarization of pro‐tumoral neutrophils, further facilitating immune evasion in solid tumors [39]. The observed alterations in dendritic cell and macrophage infiltration suggest that HPV‐positive tumors may similarly exploit these metabolic‐immune axes to maintain an immunosuppressive milieu. These transcriptional patterns imply that HPV‐positive tumors may rely on a finely tuned balance between activating antiviral responses and selectively suppressing downstream immune effectors; such a mechanism supports viral persistence while evading immune clearance. By integrating enrichment analysis, machine learning‐based feature selection, and immune cell correlation mapping, a set of immune‐evasion‐related signature genes (IFNGR1, PSMB9, and TRADD) with strong HPV dependence was identified. These genes participate in key pathways associated with antiviral defense, interferon signaling, and antigen processing, suggesting that HPV‐positive tumors may exploit these regulatory nodes to fine‐tune immune activation while enabling immune escape [40–43].

From a biological perspective, IFNGR1 encodes the ligand‐binding chain of the IFN‐γ receptor complex; its upregulation in HPV‐positive tumors may paradoxically sustain low‐level IFN‐γ signaling that promotes JAK‐STAT pathway activity while concurrently rendering cells tolerant to cytotoxic immune pressure, a mechanism described in various viral malignancies [44, 45]. TRADD, an adaptor protein linking TNFR1 to both NF‐κB activation and caspase‐mediated apoptosis, may shift the balance toward pro‐survival NF‐κB signaling rather than apoptosis when elevated in HPV‐positive cells, which aligns with the known ability of HPV E6/E7 to modulate death receptor pathways [46]. PSMB9 (also known as LMP2) encodes an immunoproteasome catalytic subunit essential for MHC class I antigen presentation; its dysregulation may impair peptide loading onto MHC‐I molecules, subsequently reducing recognition and killing by CD8+ cytotoxic T lymphocytes [47]. Collectively, these three genes may interact functionally to coordinate the suppression of cytotoxic immune responses: PSMB9 limits the breadth of viral peptides presented on the tumor cell surface, TRADD sustains NF‐κB‐mediated survival signals, and IFNGR1 modulates the tumor cell sensitivity to interferon‐mediated immune surveillance. The regulatory interaction network constructed around these genes further illuminated potential upstream transcriptional regulators and downstream immune effectors, providing a systems‐level perspective on how HPV may orchestrate immune remodeling [48].

Immune infiltration analysis further supported the hypothesis that HPV infection reshapes the CC immune microenvironment in a nonlinear and selective manner. Significantly higher infiltration levels of activated dendritic cells, plasma cells, and resting dendritic cells were observed in HPV‐positive tumors compared to HPV‐negative tumors, aligning with previous studies [49]. This pattern may indicate enhanced attempts at antigen presentation in response to persistent viral antigens, coupled with functional dysregulation that hinders effective antitumor immunity. In contrast, macrophage M0 levels were markedly elevated in HPV‐negative tumors, suggesting a comparatively immunosuppressed or undifferentiated macrophage state that may represent a distinct immune escape route independent of HPV infection. This observation corroborates previous findings, which indicated that HPV‐positive tumors consistently exhibit lower M0 macrophage levels compared to their HPV‐negative counterparts [50]. Collectively, these results reinforce the complexity of HPV‐associated immune remodeling and highlight how viral infection influences gene expression and reshapes the tumor immune ecosystem to promote immune evasion [51, 52], and further survival analysis indicates that elevated IFNGR1 expression may be associated with worse OS in HPV‐positive CC.

Additionally, the expression patterns of IFNGR1, TRADD, and PSMB9 across major cellular compartments were examined, revealing distinct distribution profiles. This supports the concept that HPV‐associated immune modulation operates at multiple cellular levels, impacting both immune regulatory nodes and intrinsic tumor antigen‐processing machinery. Complementing the transcriptomic results, in vitro experiments demonstrated different expression patterns of these genes in HPV‐positive versus HPV‐negative CC cell lines. Functional assays indicated that knockdown of IFNGR1 significantly suppressed the proliferation, migration, and invasion of HPV‐positive CC cells while promoting apoptosis. This finding positions IFNGR1 not only as a transcriptional marker of HPV‐associated immune modulation but also as a functional mediator of malignant phenotypes in HPV‐positive tumors.

Despite these strengths, several limitations warrant acknowledgment. First, while the integrated multi‐omics analyses generated robust HPV‐associated immune signatures, reliance on publicly available bulk and single‐cell datasets introduces potential biases related to clinical heterogeneity, sample processing, and incomplete annotation. Although batch effect correction was applied during the single‐cell analysis workflow, residual batch effects may persist due to discrepancies in sequencing platforms, sample processing procedures, and patient characteristics across datasets. Second, CC exhibits substantial inter‐patient and intra‐tumoral heterogeneity, potentially influencing gene expression patterns and immune cell composition. Thus, the identified signatures require further validation in larger multicenter cohorts. Additionally, while the functional relevance of IFNGR1 was confirmed in vitro, the broader regulatory network inferred from bioinformatic predictions remains to be experimentally validated as the context‐dependent nature of immune regulation may affect these interactions. Lastly, HPV genotype‐specific effects were not evaluated; distinct high‐risk subtypes may influence immune evasion differently. These limitations highlight the necessity for larger, well‐annotated cohorts and mechanistic studies to enhance the understanding of HPV‐associated immune remodeling.

## 5. Conclusion

This study integrated bulk and single‐cell transcriptomics to elucidate previously unrecognized HPV‐associated immune‐evasion signatures and regulatory networks in CC, identifying IFNGR1, TRADD, and PSMB9 as critical immune‐evasion genes. These findings advance the understanding of HPV‐mediated immune escape mechanisms and highlight IFNGR1 as a potential molecular target for therapeutic intervention in HPV‐positive CC, and further survival analysis indicates that elevated IFNGR1 expression may be associated with worse OS in HPV‐positive CC.

## Author Contributions

Na Zhang conceived and designed the research. Rui Guo conducted the experiments. Wenting He and Xia Liu analyzed the data. Rui Guo wrote the manuscript.

## Funding

This study was supported by the Key Research and Development Program of Ningxia Hui Autonomous Region (Grant 2023BEG03048).

## Disclosure

All authors read and approved the manuscript.

## Conflicts of Interest

The authors declare no conflicts of interest.

## Supporting Information

Additional supporting information can be found online in the Supporting Information section.

## Supporting information


**Supporting Information 1** Table S1: Differentially expressed genes between HPV‐positive and HPV‐negative cervical cancer samples.

**Supporting Information 2** Table S2: Human orthologs of 180 immune evasion‐related genes.

## Data Availability

The data that support the findings of this study are available from the corresponding author upon reasonable request.
